# Electrostatic wave breaking limit in a cold electronegative plasma with non-Maxwellian electrons

**DOI:** 10.1038/s41598-021-85228-z

**Published:** 2021-03-17

**Authors:** I. S. Elkamash, I. Kourakis

**Affiliations:** 1 Physics Department, Faculty of Science, Mansoura University, 35516 Mansoura, Egypt; 2 Mathematics Department, Khalifa University of Science and Technology, College of Science and Engineering, P.O. Box 127788, Abu Dhabi, UAE

**Keywords:** Physics, Plasma physics

## Abstract

A one-dimensional multifluid hydrodynamic model has been adopted as basis for an investigation of the role of suprathermal electrons on the wave breaking amplitude limit for electrostatic excitations propagating in an electronegative plasma. A three-component plasma is considered, consisting of two inertial cold ion populations of opposite signs, evolving against a uniform background of (non-Maxwellian) electrons. A kappa-type (non-Maxwellian) distribution function is adopted for the electrons. By employing a traveling wave approximation, the first integral for the fluid-dynamical system has been derived, in the form of a pseudo-energy balance equation, and analyzed. The effect of intrinsic plasma parameters (namely the ion density ratio, the ion mass ratio, and the superthermal index of the nonthermal electrons) on the wave breaking amplitude limit is explored, by analyzing the phase space topology of the associated pseudopotential function. Our results are relevant to particle acceleration in Space environments and to recent experiments based on plasma-based accelerator schemes, where the simultaneous presence of negative ions and nonthermal electrons may be observed.

## Introduction

Wave breaking is a topic of fundamental interest in various plasma based applications, including (but not limited to) particle acceleration experiments^1–3^, laser-assisted fusion schemes^4^, collisionless heating^5,6^ and heating of the solar corona^7,8^, to mention a few. The wave (amplitude) breaking limit (WBL) of a nonlinear plasma excitation (wave) represents the maximum amplitude of an electric field generated by the space-charge distribution due to the wave propagating: beyond this limit, the coherent nature of the wave is destroyed and the electromagnetic energy associated with the wave is randomly distributed over the particles, thus effectively heating the plasma^9–11^. At the wave breaking point, the fluid speed of the inertial plasma component exceeds the phase speed of the wave. A multistream flow thus develops and coherent waveforms are destroyed, hence converting localized energy (due to collective phenomena) into microscopically randomized (i.e. thermal) energy.

The presence of a fraction of negative ions in a plasma, in so called negative-ion plasmas (NIP), in addition to the positive ions needed to maintain overall charge neutrality, has been shown in a number of studies to affect the dynamical behavior quite dramatically. Negative ion plasma is not only generated in the laboratory^12–15^ but also occurs in Space, for instance in the D and F regions of the Earth’s ionosphere^16^ and in the inner coma of comet Halley^17^. NIP are utilized in industrial applications, e.g. injection of a beam to accomplish plasma heating in plasma etching^18^, in material processing^19^ and in fusion reactors^20^. These authors recently studied the effects of the kinematic viscosity and ion drag on electrostatic (ES) shocks in NIP^21^, while the effect of a negative ion beam in a collisionless, unmagnetized quantum ultradense plasma was studied in a subsequent study^22^; interestingly, the coexistence of negative and positive polarity solitary structures was predicted in the latter case.

It is by now established that particle acceleration mechanisms may lead to electron distributions with an increased relative weight of the superthermal component of the distribution function, where a power-law dependence may be observed^23^. A standard approach to take this situation into account is by adopting a so-called *kappa* distribution function. The kappa ($\kappa$) distribution was originally introduced phenomenologically to model the (non-thermal) particle distribution observed in the magnetosphere instruments onboard the OGO-1 and OGO-3 satellites^24^. The real parameter kappa ($\kappa$), after which the kappa distribution is named, is the spectral index of the distribution function: for small values of $\kappa$, distributions feature a long tail and hence a large portion of superthermal particles (the Maxwellian distribution is recovered for very large values of $\kappa$, viz. $\kappa \rightarrow \infty$). Hellberg et al.^25^ derived the generalized plasma dispersion function for electrostatic waves in kappa-distributed plasmas. Baluku et al. later modeled the propagation of dust ion-acoustic waves via a kinetic description^26^. A fluid description has been adopted to showcase the effect of a kappa-distributed background on ionic scale excitations in Ref.^27^ (also see the references therein). The effect of superthermal (kappa-distributed) electrons on multicomponent plasma expansion into vacuum was recently investigated^28^.

Akhiezer and Polovin^29^ were the first to consider a cold relativistic plasma (where the massive ions provide a fixed charge neutralizing background for electron motion), introducing the maximum (wave breaking) amplitude limit of an electron plasma (Langmuir) wave. They predicted that this amplitude limit approaches infinity as the phase velocity of the wave approached the speed of light *c*. Using a Lagrangian description, Dawson^9^ derived the WBL $E_{wb}$ limit for a cold classical (non-relativistic) plasma in a fixed ion background. Coffey^30^ later investigated thermal effects in non-relativistic plasma, by employing a one-dimensional (1D) waterbag model for the electrons: thermal pressure was, in fact, shown to reduce the WBL, in comparison with cold classical plasma. Back to the relativistic regime, for a cold plasma with immobile ions, Katsouleas and Mori analytically studied the WBL^31^; as in the nonrelativistic case^30^, they have found that including finite electron temperature suppresses WBL growth, so that is does not approach infinity as the phase speed becomes strongly relativistic, as predicted in^29^. Ionic motion in a cold relativistic electron-ion plasma was later considered by Khachatryan in an extension of earlier studies^32^. In fact, a larger ion mass (i.e. a decrease of the electron-to-ion mass ratio) was shown to decrease the WBL (amplitude limit). The WBL behavior for arbitrary phase speeds in a 1D warm relativistic electron plasma model was investigated in Ref.^33^, where the correspondence between wave breaking and background particle trapping was discussed for the first time. Using a Lagrange variable methodology, Maity et al. have introduced an exact space time-dependent solution for nonrelativistic upper hybrid oscillations at breaking point, in the presence of an inhomogeneous magnetic field^34^ and, later, of relativistic upper-hybrid oscillations in a cold homogeneous magnetized plasma^35^. Their study was later extended to cover electron and positron oscillations in a collisionless, unmagnetized, non-relativistic electron—positron-ion plasma^36^. In a recent study, Karmakar et al.^37^ adopted a travelling wave approximation and a pseudopotential formalism, to derive analytical predictions for the relativistic wave-breaking limit for the existence of the electrostatic plasma waves, in a cold relativistic electron-positron-ion plasma. They showed that adding of a fraction of massive ions in a pure electron-positron plasma leads to a reduction in the value of the maximum allowed electric field amplitude to be sustained before wave-breaking. In Ref.^38^, Karmakar *et al.* have studied the effect of an external magnetic field on the wave-breaking limit for relativistic upper-hybrid (RUH) oscillations in a cold magnetized plasma. They have shown that the wave-breaking amplitude of RUH wavepackets is suppressed (decreases) as the ambient magnetic field gets stronger. Pramanik et al. have examined the impact of the external magnetic field on phase-mixing and wave breaking phenomena, with respect to electrostatic oscillations in cold classical (nonrelativistic) electron-positron-ion plasmas^39,40^. The relevant cold fluid equations for nonrelativistic electron-positron-ion plasmas have been numerically solved to investigate the wave-breaking of a Langmuir wave and to distinguish between the predicted wave-breaking and phase-mixing time (scales)^41^. Jana et al.^42^ analytically estimated the maximum sustainable electric field amplitude associated with nonlinear relativistic electron acoustic waves in homogeneous, unmagnetized plasma in a two-electron plasma model. influence of thermal electron motion on relativistic plasma oscillations (breaking) was discussed In Ref.^43^, where it was shown that wavebreaking is suppressed entirely due to conversion of plasma oscillations into travelling waves beyond a certain electron temperature. Frolov *et al.*^44^ investigated the role of the initial electron density profile (distribution) on the breaking of nonlinear Langmuir oscillations. Using a 1D particle-in-cell (PIC) code, Rathee *et al.* explored the effect of the electron temperature and of the background inhomogeneity on the wave-breaking limits in warm, electron–ion plasmas^45^, and pinpointed the existence of a critical electron temperature beyond which wave-breaking does not occur. More recently, Adak et al. discussed the wave-breaking limit for nonlinear ES waves in a non-relativistic warm two-ion-species plasma model^46^. They pointed out that an increase in temperature leads to a decrease in the maximum amplitude of ES wavepackets, while the latter mildly increases with an increase in the ion mass ratio.

In this paper, we have undertaken an investigation of the wave-breaking amplitude of one-dimensional electrostatic waves in cold electronegative plasma in the presence of suprathermal electrons. Understanding the laws governing the dynamical evolution of self-sustained electric fields is of crucial importance for plasma-based particle acceleration schemes, as this is among the critical parameters that determine the maximum energy gain by the accelerated particles^47–49^. As we have mentioned, negative-ion plasma occurs in various environments, both in Space and in the lab, and this is always characterized by the existence of accelerated (suprathermal) electrons in the background. Our investigation outcomes will be useful in the interpretation of particle acceleration mechanisms in both laboratory and astrophysical environments.

## The fluid model

We shall now consider a collisionless, unmagnetized, homogeneous plasma containing positive ion species with mass $m_1$ and positive charge $q_1 = +z_1 e$, negatively charged—ion population, with mass $m_2$, charge $q_2 = -z_2 e$) and nonthermal electrons $n_e$ modelled by the kappa-distribution function; *e* denotes the elementary (absolute) charge, as usual. We assume that any spatial variation of the plasma state variables essentially takes place in the longitudinal direction (only), hence a 1D geometry is adopted for simplicity.

In 1D planar geometry, the fluid model equations can be written as :

1 $$\begin{aligned} \frac{\partial n_{1}}{\partial t}+\frac{\partial }{\partial x} (n_{1}u_{1})&=0 , \end{aligned}$$

2 $$\begin{aligned} m_1n_1\bigg (\frac{\partial u_{1}}{\partial t}+u_{1} \frac{\partial u_{1}}{\partial x} \bigg )&=-z_1en_1\frac{\partial \phi }{\partial x}, \end{aligned}$$

3 $$\begin{aligned} \frac{\partial n_{2}}{\partial t}+\frac{\partial }{\partial x} (n_{2}u_{2})&=0 , \end{aligned}$$

4 $$\begin{aligned} m_2n_2\bigg (\frac{\partial u_{2}}{\partial t}+u_{2} \frac{\partial u_{2}}{\partial x}\bigg )&=z_2en_2 \frac{\partial \phi }{\partial x}, \end{aligned}$$

5 $$\begin{aligned} \frac{\partial ^2 \phi }{\partial x^2}&= - \frac{1}{\epsilon _0} e \big (z_1n_1 -z_2n_2- n_e\big ) \, , \end{aligned}$$

The plasma state variables $n_j$, $u_j$ respectively indicate the number density and the flow fluid velocity, of species(s) $j=1$ (for the positive ion fluid) or $j=2$ with (for the negative ion fluid), where $m_j$ and $z_j$ respectively indicate the mass and charge state of species *j* (for $j=1$ or 2), while $\epsilon _0$ is the susceptibility of vacuum. Note the definition of the electrostatic potential $\phi$, related to the electric field by ${\varvec{E}} = - \nabla \phi$.

The density of the $\kappa$ distributed electrons is given by^50,51^:

6 $$\begin{aligned} n_e=n_{e, 0}\bigg (1-\frac{e\phi }{T_e(\kappa -\frac{3}{2})}\bigg )^{(-\kappa +\frac{1}{2})}, \end{aligned}$$

where $T_e$ is the electron temperature and $\kappa$ is the spectral index; recall that $\kappa > 3/2$ for physically realistic solutions.

For simplicity in algebraic manipulation, the model evolution equations can be rescaled as:

7 $$\begin{aligned} \frac{\partial n_{1}}{\partial t}+\frac{\partial }{\partial x} (n_{1}u_{1})&=0 , \end{aligned}$$

8 $$\begin{aligned} \frac{\partial u_{1}}{\partial t}+u_{1}\frac{\partial u_{1}}{\partial x}&=-\frac{\partial \phi }{\partial x}, \end{aligned}$$

9 $$\begin{aligned} \frac{\partial n_{2}}{\partial t}+\frac{\partial }{\partial x} (n_{2}u_{2})&=0 , \end{aligned}$$

10 $$\begin{aligned} \frac{\partial u_{2}}{\partial t}+u_{2}\frac{\partial u_{2}}{\partial x}&=\frac{Q}{\mu }\frac{\partial \phi }{\partial x}, \end{aligned}$$

11 $$\begin{aligned} \frac{\partial ^2 \phi }{\partial x^2}&= \beta n_{e}-n_{1}+\delta n_{2} \, , \end{aligned}$$

where all quantities are dimensionless. The normalized electron density reads^50,51^

12 $$\begin{aligned} n_e=\bigg (1-\frac{\phi }{\kappa -\frac{3}{2}}\bigg )^{-\kappa +\frac{1}{2}} \, . \end{aligned}$$

The closed system of Eqs. (7)–(12) will form the basis of our analysis to follow. We have defined the dimensionless quantities: $\mu =\frac{m_2}{m_1}$, $Q=\frac{q_2}{q_1}$ where $q_1=z_1 e$, $q_2=z_2e$.

In the above fluid equations, time *t* and space *x* have respectively been normalized by (the positive ion plasma period) $\omega _{p,1}^{-1}=(z_{1}^{2} e^{2}n_{1, 0}/\epsilon _0 m_{1})^{-1/2}$ and by (the positive ion Debye length) $\lambda _{D, 1}=\left( \epsilon _0 k_{B}T_{e}/z_{1}e^{2}n_{1, 0}\right) ^{1/2}$. The number density $n_j$ was normalized by the respective unperturbed number density $n_{j0}$ (for each fluid; viz. $j= e, 1, 2$ for electrons, ions 1 and ions 2), while the fluid speed $u_j$ variable(s) was (were both) normalized by the characteristic speed $c_s = (z_1 K_B T_e/m_1)^{1/2}$ . The electrostatic potential $\phi$ is normalized by $k_B T_e/e$. We retain in the following the definition of the parameters

13 $$\begin{aligned} \delta = \frac{z_2 n_{20}}{z_1 n_{10}} \qquad \mathrm{and} \qquad \beta =\frac{n_{e0} }{z_1n_{10}}\, , \end{aligned}$$

i.e. the negative-to-positive ion density ratio and the electron-to-positive-ion density ratio, respectively. At equilibrium (where $n_{j,0}=1, \quad \forall j$), overall charge neutrality dictates:

14 $$\begin{aligned} \beta = 1 - \delta \, . \end{aligned}$$

## Travelling wave approximation: pseudopotential formalism

Anticipating stationary-profile solutions in a reference frame moving at $M(=\frac{V_{ph}}{c_s}$) where $V_{ph}$ denotes the phase speed of the electrostatic solitary wave and $c_s$ is the sound speed (reference value, in *e-i* plasmas), we shall express all state variables as functions of a single moving coordinate $\xi = x- M t$, viz.

$$\begin{aligned} \frac{\partial }{\partial t}=- M \frac{\partial }{\partial \xi }, \qquad \frac{\partial }{\partial x}=\frac{\partial }{\partial \xi } \, . \end{aligned}$$

Time variation is ignored, since stationary-profile solutions are expected. Any stationary solution (in the moving frame) will break down once the condition of existence of such solutions is violated, i.e. as soon as large-scale particle trapping occurs. In classical plasma theory of electrostatic solitary waves^52,53^ *M* is termed the “Mach number”, in analogy to sound waves in air, which are modeled by similar equations.

Equations (7)–(11) are thus transformed into a system of (coupled) ordinary differential equations (ODEs):

15 $$\begin{aligned} \frac{\partial }{\partial \xi }[n_1(u_1-M)]&= 0, \end{aligned}$$

16 $$\begin{aligned} \frac{\partial }{\partial \xi }\big [\frac{1}{2} u_1(u_1-2M)+\phi \big ]&= 0, \end{aligned}$$

17 $$\begin{aligned} \frac{\partial }{\partial \xi }\big [n_2(u_2-M)\big ]&= 0, \end{aligned}$$

18 $$\begin{aligned} \frac{\partial }{\partial \xi }\big [\frac{1}{2} u_2(u_2-2M)-\frac{Q}{\mu }\phi \big ]&= 0,\end{aligned}$$

19 $$\begin{aligned} \frac{\partial ^2 \phi }{\partial \xi ^2}+n_1-\delta n_2-\beta n_e&=0 . \end{aligned}$$

After some manipulation, Eqs. (15)–(19), the dimensionless velocity and density variables of the fluids read:

20 $$\begin{aligned} u_1=&M\left( 1-\sqrt{1-\frac{2\phi }{M^2}}\right) \nonumber \\ u_2=&M\left( 1-\sqrt{1+\frac{2Q\phi }{\mu M^2}}\right) \nonumber \\ n_1 =&\frac{1}{\sqrt{1-\frac{2\phi }{M^2}}},\nonumber \\ n_2 =&\frac{1}{\sqrt{1+\frac{2Q\phi }{\mu M^2}}}. \end{aligned}$$

The plasma state variables $n_j$ and $u_j$ should obviously be real. The reality requirement of the positive ion fluid speed and density imposes the constraint $0< \phi < \frac{1}{2}M^2$. Physically speaking, as the value of the ES potential $\phi$ approaches the critical value $\phi _{cr, p}$ $(=\frac{1}{2}M^2)$, the peak fluid speed approaches the phase speed of the wave and the (positive) ion density becomes infinite (infinite compression limit). The analogous expression for the second (negative) ion fluid reads: $\phi _{cr,n} = -\frac{\mu }{2 Q}M^2< \phi < 0$. Note the subscript “cr” (for “critical”), denoting the critical values for the positive (*p*) or for the negative (*n*) ions, respectively. While both limits should be considered in a given plasma (due to the simultaneous occurrence of the positive and negative ion fluids), hence both negative and positive displacements from the equilibrium state are bounded (in absolute value), it is clear that the topology of the energy curve will determine the maximum value of the wave energy (i.e. one only—and not *both*—of these limits may be accessible by the dynamics). This point will be further elaborated upon in the following.

Using the latter two expressions to eliminate $n_j$ in Eq. (19), the system of equations (15)–(19) can be reduced to a second-order differential equation for $\phi$ in the form:

21 $$\begin{aligned} \frac{\partial ^2 \phi }{\partial \xi ^2}+ \frac{\partial U}{\partial \xi }=0 \, , \end{aligned}$$

where the nonlinear function $U(\phi )$ is given by:

22 $$\begin{aligned} U(\phi )=&M^2\Bigg (1-\sqrt{1-\frac{2\phi }{M^2}}\Bigg )+\delta \frac{\mu }{Q} M^2\Bigg (1-\sqrt{1+\frac{2Q\phi }{\mu M^2}}\Bigg ) \nonumber \\&+\beta \Biggl [1-\bigg (1-\frac{\phi }{\kappa -\frac{3}{2}} \bigg )^{-(\kappa -\frac{3}{2})}\Biggr ] \, . \end{aligned}$$

Note that Eq. (21) has the form of a 1D equation of motion for a (unit mass) particle moving in a field with potential $U(\phi )$; the values $\phi$, $\frac{\partial \phi }{\partial \xi }$, and $\frac{\partial ^2 \phi }{\partial \xi ^2}$ represent the displacement (from equilibrium), the velocity and the acceleration, respectively, of this fictitious particle; the independent variable $\xi$ represents “time” in this pseudomechanical analogy. The pseudopotential $U(\phi )$ is equal to zero at a point $\phi = 0$ where the electric field reaches a local maximum. The dynamical features of the electric field associated with the propagating wave can therefore be determined by studying the topology of the pseudopotential function $U(\phi )$.

For $\delta \rightarrow 0$, and $\kappa \rightarrow \infty$, we get:

23 $$\begin{aligned} U(\phi )=\Bigg (1-e^\phi \Bigg )+M^2\Bigg (1-\sqrt{1-\frac{2\phi }{M^2}}\Bigg ) \end{aligned}$$

which recovers the known form of the pseudopotential curve for cold positive ions plasmas with Maxwellian electrons^52,53^. The latter expression therefore describes large-amplitude (nonlinear) periodic ion-acoustic waves in cold electron-ion plasmas (i.e. in the absence of negative ions).

On the other hand, for $\delta \rightarrow 1$, $Q \rightarrow 1$ and $\mu \rightarrow 1$, we obtain:

24 $$\begin{aligned} U(\phi )=M^2\Bigg (1-\sqrt{1-\frac{2\phi }{M^2}}\Bigg )+ M^2\Bigg (1-\sqrt{1+\frac{2\phi }{M^2}}\Bigg ), \end{aligned}$$

which is the expression of the pseudopotential in the cold pair-ion fluid plasma model^54,55^. Expression (24) therefore describes large-amplitude (nonlinear) periodic ion-acoustic waves in pure pair-ion—e.g. fullerene—plasmas (i.e. in the absence of electrons).

Carrying out an integration in Eq. (21), the first integral of motion can be obtained in the form:

25 $$\begin{aligned} \frac{1}{2} \biggl (\frac{\partial \phi }{\partial \xi }\biggr )^2 + U(\phi )=I, \end{aligned}$$

i.e.

26 $$\begin{aligned} \frac{\partial \phi }{\partial \xi }=\pm \sqrt{2(I-U(\phi ))} \, , \end{aligned}$$

where *I* is an (arbitrary) integration constant. Eq. (25) represents a conservation law of a fictitious particle with unit mass, where the first term on the left hand side represents the kinetic energy while the second term is the potential energy, hence *I* can be identified as the total pseudo-mechanical-energy of the particle. Recalling that the self-generated electric field is related to the electrostatic potential $\phi$ as ${\varvec{E}} = - \nabla \phi$ (i.e. in 1D geometry $E = - \partial \phi /\partial \xi$ in the moving frame), we see that the right-hand side of the latter algebraic expression provides the range of value(s) to be attained by the $E-$field.

The occurrence of plasma waves within the above model relies on ensuring, within the analytical model, the reality of the potential $U(\phi )$; this fact imposes constrains on the permitted values the pseudopotential $U(\phi )$ and hence on the values of the electrostatic potential $\phi$ supported by the plasma waves. Therefore, the amplitude of the electric field will not achieve an arbitrarily large amplitude due to the limitations on the allowed values of $U(\phi )$. To determine the maximum attainable electric field associated with the plasma waves, i.e. the wave-breaking limits for the electrostatic waves, we must consider the maximum “allowed” values of $U(\phi )$ (say, $U_{max}$). Therefore, our fictitious particle will vibrate inside the potential well with the highest possible amplitude determined by the largest allowed value of $U(\phi )$, i.e. $\phi _{max}$. In simple words, the dynamics will not “visit” any values of $U(\phi )$ above $U_{max}$ (calculated at $\phi _{max}$), where the integration constant *I* in the Eq. (25) determines the maximum energy, i.e. $I = U(\phi =\phi _{max})=U_{max}$. For a given (prescribed) value of *I*, the wave-breaking amplitude $E_{wb}$ of the electric field to be supported in the plasma reads:

27 $$\begin{aligned} E_{wb}=\sqrt{2U_{max}} \end{aligned}$$

where $U_{max}$ is the maximum allowed value of $U(\phi )$, $E_{wb}$ is the maximum electric field of the sustainable wave amplitude beyond which the wave is broken and the wave coherence is destroyed. Also, at the critical value of the potential $U(\phi )$, the occurrence of wave breaking is associated with the nonreality of the plasma density, which physically indicates the infinity density compression and density gradients. At the onset of wave breaking, wave coherence is destroyed and the wave energy is converted to random particle energy leading to particle acceleration.

From the expression of $n_j$, we can find that the number density is real only in the range $\phi _{cr,n} \le \phi \le \phi _{cr,p}$ where $\phi _{cr,n}=-\frac{\mu }{2 Q}M^2$ and $\phi _{cr,p}=\frac{1}{2}M^2$ represent the lower and upper bounds for the wave breaking field, respectively. Our expression of $U(\phi )$ indicates that it is not real in the whole parameter space of our system but only in the constrained values of the electrostatic potential $\phi$, i.e., $U(\phi )$ is real only in the domain $[\phi _{cr,n}$, $\phi _{cr,p}]$, elsewhere $U(\phi )$ is not real and can not support the nonlinear periodic plasma waves. Therefore, on the negative side, beyond $\phi _{cr,n}$ and on the positive side beyond $\phi _{cr,p}$, the solution does not support the existence of nonlinear periodic ion acoustic waves.

On the positive $\phi$ side, periodic solutions are possible upto $U_{max,p}$ calculated at $\phi =\phi _{cr,p}$, where $U_{max,p}=U(\phi =\phi _{cr,p})$. Consequently, the wave-breaking amplitude reads

28 $$\begin{aligned} E_{wb,p}=\sqrt{2 U_{max,p}} \, . \end{aligned}$$

On the negative $\phi$ side, periodic solutions are possible upto $U_{max,n}$ calculated at $\phi =\phi _{cr,n}$, where $U_{max,n}=U(\phi =\phi _{cr,n})$.

29 $$\begin{aligned} E_{wb,n}=\sqrt{2 U_{max,n}} \end{aligned}$$

As a consequence, the maximum permissible value of $U(\phi )$, i.e. $U_{max}=\min \{U_{max,p},U_{max,n}\}$ and hence the actual wave-breaking amplitude limit will be $E_{wb}=\min \{E_{wb,p}, E_{wb,n}\}=\sqrt{2 U_{max}}$. Accordingly, the electrostatic potential will take values between two extrema, viz. $\phi _{min}< \phi < \phi _{max}$, whose values satisfy $U(\phi _{min}) = U(\phi _{max}) = I$. Note that $U(\phi )$ is not an even function in its argument, i.e. the curve will *not* be symmetric, in general. (To see this, note that $U(-\phi ) = U(\phi )$, with the sole exception of pair-ion plasma—described by Eq. (24) above—which is not our focus in this study and will not be considered further.) As a consequence, anharmonic (nonlinear) periodic electrostatic waves will *not* be symmetric in the ES potential, i.e. $\phi _{min} \ne \phi _{max}$ in general, nor is any symmetry expected in the electric field either.

In the textbook case of cold positive ions with Maxwellian electrons in an e-i plasma, i.e. with $\delta = 0$ and $\kappa \rightarrow \infty$, we obtain:

30 $$\begin{aligned} E_{wb,p}= \bigg [2 \big (1-e^{\frac{M^2}{2}}+M^2\big )\bigg ]^{1/2} \, . \end{aligned}$$

Considering a different limit now, that of cold pair-ion plasmas. i.e. $\delta = Q = \mu = 1$, one is led to:

31 $$\begin{aligned} E_{wb,p}=1.08239M. \end{aligned}$$

which agrees precisely with Eq. (17) in Ref.^46^ with $\mu = M=1$.

## Wave breaking limit parametric analysis

We shall now discuss the impact of various plasma parameters, such as the superthermality index $\kappa$, the ion density ratio $\delta$, the ion mass ratio $\mu$, and the phase speed (“Mach number”) *M* on the pseudopotential profile $U(\phi )$ and on the maximum electric field (wave breaking limit) $E_{wb}$.

**Figure 1:**
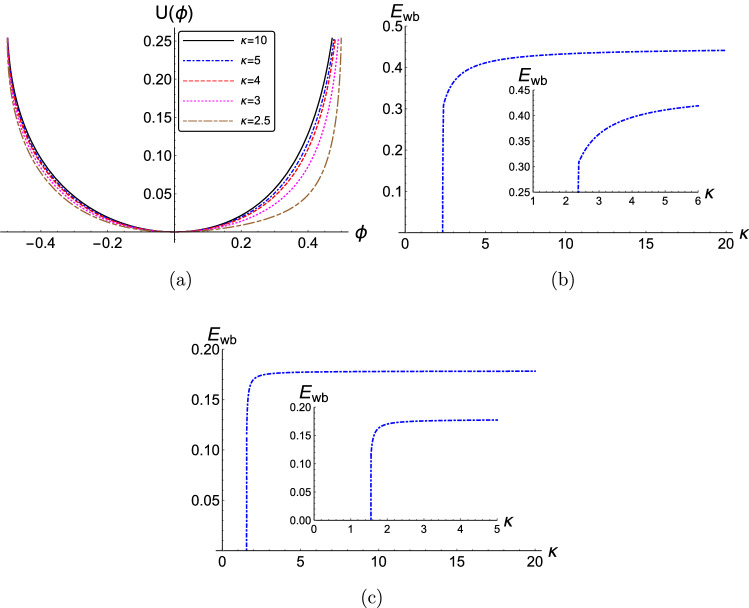
(Color online) (**a**) The effect of the variation of the superthermality index $\kappa$ on the pseudopotential $U(\phi )$ is depicted versus the electrostatic potential $\phi$ for $M=1$. (**b**) The variation of the wave-breaking limit (electric field amplitude) $E_{wb}$ is depicted versus the value of the spectral (superthermality) index $\kappa$. (**c**) As the previous panel, but shifting the value of *M* to 0.3 (all other parameter values being the same). The parameter values adopted in these graphs are: $\delta =0.5$, $\mu =1$, and $Q=1$. The numerical values adopted here, as imposed by the maximum allowed value(s) of $U(\phi )$, i.e. $U_{max} = I$, are presented in Table I (see Online Appendix A).

The influence of superthermal particles (manifested via the superthermality index $\kappa$) on the pseudopotential $U(\phi )$ profile is investigated in Fig. 1a. We note that the width of the pseudopotential curve (*U*) on the positive side (of the electrostatic potential $\phi$) increases as the superthermal index $\kappa$ decreases (implying a stronger deviation from the Maxwellian), while it remains practically unaffected on the negative side of $\phi$. Figure 1b depicts the wavebreaking limit $E_{wb} = \min \{E_{wb,p},E_{wb,n}\}$. Assuming an increased number of particles in the suprathermal region of the electron distribution, i.e. a lower value of $\kappa$, clearly results in a lower value of the maximum amplitude (wave-breaking limit). The physical origin of this behavior can be sought in the impact of the superthermality index $\kappa$ on the charge (Debye) screening mechanism. It is known that the charge screening length is shorter in plasmas where the electron background deviates from the Maxwell-Boltzmann, i.e. $\lambda _{D,\kappa } < \lambda _{D, Maxwell}$; see e.g.^27,56^. This is certainly correlated physically with the behavior of the wavebreaking limit (as it varies with $\kappa$), as observed here. For comparison, localized waves in the same model (i.e. supersonic solitary waves, associated with localized S-shaped bipolar $E-$field structures) witness an *increase* in their amplitude, for lower $\kappa$ (i.e. for stronger electron super-thermality)^51,56^. In our case here (for anharmonic i.e. nonlinear periodic waves), it appears that the maximum amplitude is suppressed by small values of $\kappa$, as shown in Fig. 1b. (Recall that values of $\kappa$ between, say, $\simeq 2$ and $\simeq 6$ are characteristic—and actually ubiquitous—in Space environments.) Note that an asymptotic value (e.g. $E_{wb, \infty } \simeq 0.448$ in Fig. 1b) is reached for large values of $\kappa$, since the electron distribution is practically of Maxwell-Boltzmann type at those values.

It is interesting to point out the existence of a cutoff in $\kappa$, for instance below $\kappa \approx 2.2$ in Fig. 1b, below which waves cannot propagate. This is due to the fact that the quantity under the square root in (27)–(29) above (27)–(28) may take negative values for certain combinations of parameter values, thus rendering $E_{wb}$ imaginary. Assuming fixed values of $\delta$ and $\mu$, the cutoff value for $\kappa$ is actually a function of *M*, hence it will depend on the phase speed. Notice e.g. the difference between Fig. 1b,c, which differ only in the value of *M*. This means that different values of $\kappa$ affect the phase speed (range of values). A strong deviation from the thermal (Maxwell-Boltzmann) picture may actually even prevent the wave from occurring; cf. Fig. 1b,c. This is reminiscent of the properties of dispersive (linear) waves in kappa-distributed plasmas^27^ and, again, is a manifestation of the effect of suprathermal particlaes on the Debye screening mechanism.

**Figure 2:**
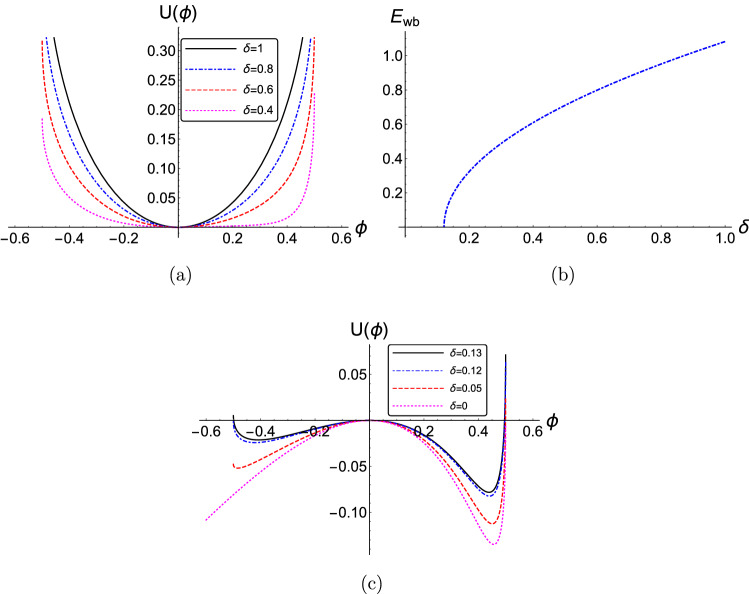
(Color online) (**a**) The effect of the variation of the negative-to-positive ion density ratio $\delta$ on the pseudopotential $U(\phi )$ is depicted, versus the electrostatic potential $\phi$. (**b**) The variation of the minimum normalized wave-breaking electric field amplitude $E_{wb}$ versus the negative ion density ratio $\delta$. (**c**) The pseudopotential $U(\phi )$ is depicted for different values of $\delta$ near the cutoff point. The parameter values are: $\kappa =2.5$, $\mu =1$, $Q=1$ and $M=1$. The numerical values adopted here, as imposed by the maximum allowed value(s) of $U(\phi )$, i.e. $U_{max} = I$, are presented in Table II (see Online Appendix A).

Figure 2a shows the impact of the concentration of the negative ion density $\delta$ (for fixed—arbitrary—values of $\kappa$, *Q*, $\mu$ and *M*), on the profile of the Pseudopotential $U(\phi )$. The pseudopotential well becomes wider as the negative-to-positive ion density ratio $\delta$ decreases. Actually, the pseudopotential becomes more asymmetric with decreasing $\delta$ (so so will the wavepacket form be expected to be). The electric field (maximum) amplitude increases with $\delta$. Interestingly, the electric field limit $E_{bw}$ is zero below $\delta \approx 0.12$. This is due to the topology of the curve $U = U(\phi )$—see e.g. Fig. 2c—that actually acquires negative values below that point (actually, near $\delta \simeq 0.12$ in Fig. 2c). On the opposite trend, no waves will exist below a certain threshold for $\delta$. As discussed above, this will be a matter of balance among the values of $\delta$, *M*, $\mu$ and $\kappa$.

We see in Fig. 2b that a higher concentration of negative ions (i.e. a higher value of $\delta$) enhances the electric field (maximum amplitude) $E_{wb}$. In a picture analogous to that of Debye screening in e-i plasmas, the reason for this enhancement may lie in the increase of the Debye screening length due to the presence of negative ions in the plasma, which agrees with the outcome of linear analysis^57^. This enables higher E-field amplitudes to be reached within the extent of the Debye (shielding) sphere.

**Figure 3:**
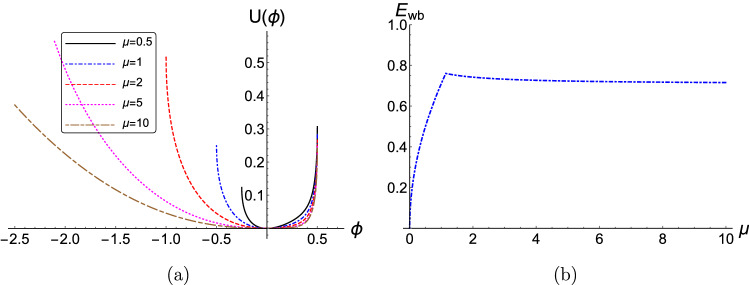
(Color online) (**a**) The effect of the variation of the ion mass ratio $\mu = m_2/m_1$ on (**a**) The pseudopotential $U(\phi )$ is depicted, versus the electrostatic potential $\phi$. (**d**) The variation of the minimum normalized wave-breaking electric field amplitude $E_{wb}$ versus the ion mass ratio $\mu$. The parameter values are: $\delta =0.5$, $\kappa =2.5$, $Q=1$ and $M=1$. The numerical values adopted here, as imposed by the maximum allowed value(s) of $U(\phi )$, i.e. $U_{max} = I$, are presented in Table III (see Online Appendix A).

The role of the ion mass ratio $\mu = m_2/m_1$ on the pseudopotential shape $U(\phi )$, and on the associated wave-breaking limit $E_{wb}$ is investigated in Fig. 3. The width of the pseudopotential remains practically unaffected on the positive side of $\phi$, while it increases dramatically on the negative side (of $\phi$) as the mass ratio $\mu$ increases, as seen in Fig. 3a.

From Fig. 3b, the maximum allowed electric field amplitude $E_{wb}$ increases sharply for $\mu \le 1$ and reaches its maximum $E_{wb}=0.71$ at $\mu =1$, i.e. pair-ion plasmas, in agreement with Eq. (11) and Fig. 2 in Ref.^32^ and with Fig. 3 in Ref^46^. Two extreme cases are worth discussing, physically. As the mass ratio $\mu \rightarrow 0$, i.e. as $m_1 \gg m_2$, the maximum allowed electric field $E_{wb}$ approaches zero. On the other hand, as the mass ratio $\mu \rightarrow \infty$, i.e. $m_2 \rightarrow \infty$, the maximum allowed electric field $E_{wb}$ has a horizontal asymptote, i.e. $\mu \rightarrow 0, \infty$, as seen in Fig. 3b. Therefore, for large values of $m_2 \gg m_1$, the maximum allowed electric field $E_{wb}$ approaches a fixed value, e.g. $E_{wb, (m_2 \gg m_1)} \approx 0.707$ (for $\delta =0.5$, $\kappa =2.5$, $Q=1$ and $M=1$) in our Fig. 3b. A qualitative explanation of these two extreme behaviors could be that when the inertia of one of the two plasma components becomes $\infty$, that species becomes stationary (immobile) and does not contribute much to the electric field; this leads to a decrease in the maximum electric field. Apparently, the main driver in this process will be the negatively charged components. As $\mu \rightarrow 0$ ($m_1 \gg m_2$), the positive species is practically stationary, and there is no contribution to $E_{wb}$ from the positive ions. On the other hand, as $\mu \rightarrow \infty$ ($m_2 \gg m_1$), the negative ion species becomes stationary, so there is no contribution to $E_{wb}$ from the negative ions but there is still a significant contribution from the electrons. It can be noted that the case $\mu > 1$ (i.e. heavier negative ions) was not covered in the studies by Khachatryan^32^ or Adak et al.^46^. The extreme case $\mu \rightarrow \infty$ (ultralarge mass negative ions) in particular could be very important, as it actually represent the situation where the secondary species is dust grains, in a dusty (complex) plasma.

**Figure 4:**
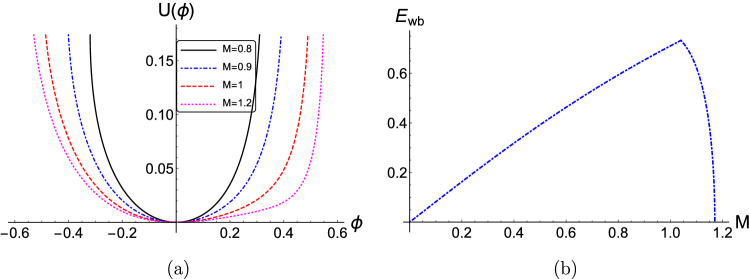
(Color online) (**a**) The effect of the variation of the phase speed (Mach number) *M* on the pseudopotential $U(\phi )$ is depicted, versus the electrostatic potential $\phi$. (**b**) The variation of the minimum normalized wave-breaking electric field amplitude $E_{wb}$ versus the Mach number *M*. The parameter values are: $\delta =0.5$, $\kappa =2.5$, $\mu =1$ and $Q=1$. The numerical values adopted here, as imposed by the maximum allowed value(s) of $U(\phi )$, i.e. $U_{max} = I$, are presented in Table IV (see Online Appendix A).

For fixed $\delta =0.5$, $\kappa =2.5$, $\mu =1$ and $Q=1$, the pseudopotential $U(\phi )$ becomes wider with increasing values of the phase speed (Mach number *M*), as observed in Fig. 4a. As *M* increases, the wave-breaking limit $E_{wb}$ increases upto 1 (one, representing the ion sound speed in scaled units) and then decreases sharply thereafter, as seen from Fig. 4b. analytically speaking, the sharp angle separating the two regions is due to the fact that the two limits given in Eqs. (28)–(29) above exchange their relative ordering at this point. (Recall that only the lower value is relevant, in the dynamics, as explained above).

## Conclusions

In this article, we have relied on a multifluid plasma model, to study the dynamics of a plasma consisting of positive ions, negative ions and nonthermal (non-Maxwellian, kappa-distributed) electrons. We have derived a first integral for the system describing stationary profile excitations at a moving reference frame. Explicit analytical relations for the evolution of the electrostatic potential and of the associated electric field have been obtained. Considering anharmonic (nonlinear) periodic waveforms (wavepackets), we have determined the wave-breaking ($E-$field amplitude) limit, both analytically and numerically, and have discussed its parametric dependence on the intrinsic plasma (configuration) parameters.

The maximum electric field (amplitude) was shown to decrease monotonically with an increase in the suprathermal electron component, i.e. for lower values of the spectral index $\kappa$: weaker electrostatic wavepackets are thus expected to occur in strongly non-Maxwellian plasmas.

The presence of the negative ion component clearly leads to an increase in the wavebreaking limit and hence the maximum allowed $E-$field amplitude. This effect is less pronounced for heavier negative ions (i.e. for larger negative ion mass $m_2$, as compared to $m_1$).

Finally, the wavebreaking limit increases for values of the phase speed not exceeding the (*e-i* plasma) characteristic sound speed ($c_s \sim (k_B T_e/m_1)^{1/2}$), hence faster $E-$field waveforms will be larger below that point. The opposite trend is witnessed above that point ($c_s$), where faster $E-$field wavepackets may be weaker.

The wave-breaking amplitude determines the maximum energy gain of energetic particles, both in Space (where particle may be accelerated via various mechanisms e.g. by cosmic rays) and in plasma-based particle acceleration schemes in the laboratory. The results presented in this article should therefore contribute towards an improved understanding of the dynamics of electrostatic disturbances in particle acceleration scenaria in plasma environments where non-Maxwellian electrons and negative ions may coexist.

It may be added, for rigor, that the electron inertia has been neglected in our model (a standard assumption, in account of the large mass disparity between the electrons and the—much heavier—ions). As a consequence, the model adopted in this article is adequate for the description of *acoustic* electrostatic wavepackets in a plasma (such as ion-acoustic waves, for instance) but fails to properly account for electron plasma waves (Langmuir waves): indeed, these are associated with a different dispersion law, namely characterized by a finite angular frequency (and an infinite phase speed but a zero group velocity) in the long wavelength limit, thanks to the electron inertia. Contrary to this picture, the wave’s phase speed has a finite value everywhere (including the infinite wavelength limit).

## Supplementary Information

Supplementary Information
