# Supplementary material for: Electrostatic wave breaking limit in a cold electronegative plasma with non-Maxwellian electrons
Source: Sci Rep. 2021 Mar 17;11:6174. doi: 10.1038/s41598-021-85228-z (PMC7969780; doi:10.1038/s41598-021-85228-z)
Supplement: Supplementary file 1 — Supplementary Information [file 41598_2021_85228_MOESM1_ESM.pdf]

A. Tables for the numerical values of maximum allowed value U_{max}

κ	10	5	4	3	2.5
$I = U_{max}$	0.278198	0.272459	0.268817	0.261027	0.252453

TABLE I: The numerical values of the upper bound (maximum allowed value) of $U(\phi)$, i.e. $U_{max} = I$, are listed for the set of values of the index κ considered in Fig. 1(a). (The remaining parameter values are as mentioned in the caption.)

δ	1	0.8	0.6	0.4
$I = U_{max}$	0.585786	0.452453	0.31912	0.185786

TABLE II: The numerical values of the upper bound (maximum allowed value) of $U(\phi)$, i.e. $U_{max} = I$, are listed for the set of values of the negative-to-positive ion density ratio δ considered in Fig. 2(a). (The remaining parameter values are mentioned in the figure caption.)

μ	0.5	1	2	5	10
$I = U_{max}$	0.125255	0.292893	0.275255	0.261387	0.255956

TABLE III: The numerical values of the upper bound (maximum allowed value) of $U(\phi)$, i.e. $U_{max} = I$, are listed for the set of values of the ion mass ratio μ considered in Fig. 3(a). (The remaining parameter values are mentioned in the figure caption.)

M	0.8	0.9	1	1.2
$I = U_{max}$	0.176115	0.213615	0.252453	0.332835

TABLE IV: The numerical values of the upper bound (maximum allowed value) of $U(\phi)$, i.e. $U_{max} = I$, are listed for the set of values of the Mach number M considered in Fig. 4(a). (The remaining parameter values are mentioned in the figure caption.)
